# Development of a murine model of oral carcinogenesis: an accelerated tool for biomarker and anti-tumour drug discovery

**DOI:** 10.3332/ecancer.2022.1413

**Published:** 2022-06-15

**Authors:** Sofia Ali Syed, Muhammad Asif Qureshi, Saeed Khan, Rajesh Kumar, Yusra Shafique, Bilal Ahmed Khan, Jawad Safdar

**Affiliations:** 1 Department of Oral Pathology, Dr. Ishrat-ul-Ibad Khan Institute of Oral Health Sciences, Dow University of Health Sciences, Karachi 74200, Pakistan; 2 Department of Pathology, Dow International Medical College, Dow University of Health Sciences, Karachi 74200, Pakistan; 3 Department of ENT, Dr. Ruth K.M. Pfau, Civil Hospital, Dow University of Health Sciences, Karachi 74200, Pakistan; 4 Department of Pathology, Dow Medical College, Dow University of Health Sciences, Karachi 74200, Pakistan; 5 Department of Oral & Maxillofacial Surgery, Dow Dental College, Dow University of Health Sciences, Karachi 74200, Pakistan; a https://orcid.org/0000-0002-8370-1069

**Keywords:** carcinogenesis, 9,10-dimethyl-1,2-benzanthracene, mice, squamous cell carcinoma, tumour burden

## Abstract

Oral squamous cell carcinoma (OSCC) is the most common cancer in Pakistani men and the second most common cancer in women. The objective of our study was to devise a novel accelerated murine model of oral carcinogenesis that can be exploited as a tool to investigate the cancer circuitry involved in OSCC and to identify molecules of diagnostic, therapeutic and prognostic significance. A total of 40 healthy male, 6–8 weeks old, 22 ± 2 gram, Naval Medical Research Institute (NMRI) outbred strain mice were recruited in the experiment. NMRI mice are commonly used for animal experiments in various fields of biology and for drug toxicity. Of these, 25 mice underwent the oral carcinogenesis regimen via topical application of 0.5% 9,10-dimethyl-1,2-benzanthracene (DMBA) on the lower left lip for a maximum of 20 weeks and 15 mice were used as controls (without the carcinogenic regimen). Exophytic tissue masses were harvested, fixed in 10% formalin and stained with haematoxylin and eosin (H&E) for microscopic diagnosis. Additionally, the expression levels of CK 5/6, p53 and Ki-67 were investigated using immunohistochemistry. Of the 25 mice which underwent the carcinogenic regimen, 21 developed moderately differentiated squamous cell carcinoma and 1 showed dysplastic features with foci of invasion. Three mice were found dead with lesion(s). CK 5/6 showed strong positivity (100%) and p53 and Ki-67 showed patchy (<30%) strong positivity in OSCC, suggesting the similarity of our model to human OSCC. We present an accelerated, close-to-human carcinogenesis, model of oral carcinogenesis using DMBA in NMRI mice that can be exploited to study the pathogenesis of oral squamous cell carcinoma and subsequently devise immunotherapy or targeted therapy.

## Introduction

Lip and oral cavity cancer (C00-C06) is the most common cancer in Pakistani men and the second most common cancer in women [1–3]. Moreover, treatment options for these tumours are largely limited to conventional surgical, chemo/radiotherapeutic (cisplatin, fluorouracil) regimens. Despite a major tumour burden in Pakistan, this tumour type is under-researched in the country [4].

Oral carcinogenesis involves the accumulation of various genetic aberrations, including DNA mutation, epigenetic changes (DNA methylation) and chromosomal aberrations. More recently, *NOTCH1, PIK3CA, AKT, PTEN* and *TRIM21* genomic changes have been identified as actionable genetic changes in oral cancers [5–7]. While chemotherapeutic regimens (cisplatin with or without fluorouracil and docetaxel) are most commonly used for the treatment of oral squamous cell carcinoma (OSCC), patients with prolonged chemotherapy develop a relapse due to chemoresistance [8]. Therefore, in order to identify relevant markers for early tumour detection and their response to chemotherapy and prognostic outcomes, it is highly relevant to undertake extensive research on this tumour type.

Diagnostic and therapeutic biomolecules can be investigated *in vivo* as well as *in vitro*. *In-vivo* models resemble more closely the complex biological environment and therefore are the mainstay of preclinical trials. For oral carcinogenesis, various murine models have been described and reviewed earlier, such as the 4-nitroquinolone 1-oxide (4NQO) model, the 9,10-dimethyl-1,2-benzanthracene (DMBA)-induced hamster cheek-pouch carcinogenesis model and the genetically modified mouse models of oral squamous cell carcinoma [9]. However, oral carcinogenesis models described in the literature did not objectively look into the number of days taken for mice to develop tumour(s). Moreover, the majority of the studies were performed using hamsters and rats. There is no anatomic counterpart of the buccal pouch in humans. Moreover, the buccal pouch epithelium is thinner in hamsters compared to mice and humans [10]. It, therefore, becomes highly relevant to modulate/tweak the existing carcinogenic regimens to develop/establish a novel close-to-human cancer murine model of accelerated oral carcinogenesis with high penetrance and replicability.

In the study presented herein, we describe an accelerated close-to-human cancer murine model of oral carcinogenesis that can be exploited not only to understand the mechanisms involved in oral carcinogenesis but also to investigate various molecular players of diagnostic, prognostic and therapeutic significance.

## Methods

A total of 40 healthy immunocompetent males, 6–8 weeks old, 22 ± 2 gram, NMRI outbred strain of laboratory mice were purchased from the Department of Animal Sciences, Dow University of Health Sciences (DUHS). All mice (5 mice/cage) were transferred to the animal testing facility and were given a week’s acclimatisation to the facility with 26 ± 2°C temperature, 55% humidity and 12-hour light/dark cycles with food and water *ad libitum*. Mice were housed in standard murine cages and with standardised bedding, food and water supply. Bedding was changed either weekly or as required. Of the 40 mice, a total of 25 were subjected to the accelerated carcinogenic regimen, while 15 mice were used as controls. The study was approved by the Institutional Ethical Review Board for Animal Research and Ethics (Ref# AR.IRB-013/DUHS/Approval/2018/020). All animal experiments were performed at the animal testing facility of DUHS under the supervision of internationally licensed personnel trained and licensed as per the Animals (Scientific Procedures) Act 1986.

The induction of oral carcinogenesis was performed as previously published [11]. Briefly, oral carcinogenesis was induced in the lower left lip of the mice via topical application of 0.5% 9,10-dimethyl-1,2-benzanthracene (DMBA; Sigma-Aldrich). The 0.5% DMBA solution was prepared by diluting 100 mg of DMBA in 20 ml of acetone at room temperature. The DMBA application was performed twice every alternate day on the lower left side of the lip with a sable brush (no. 0) for a maximum 20 weeks. The mice were inspected daily. Additionally, the weights of the mice were recorded weekly. The mice were culled if any evidence of sickness was found or if the study end point was reached, whichever was earlier.

The collected tissues were fixed in 10% formalin, followed by tissue processing in an automated tissue processor, tissue embedding in paraffin wax moulds/blocks and sectioning at 4 µm thickness. Tissue sections were subsequently stained with haematoxylin and eosin (H&E). All slides were examined and analysed under a light microscope (Motic, BA310E; USA) by a consultant histopathologist (blinded for the samples) for identification of tumour cells. Images were captured using a camera (Motic Images Plus 3.0v; USA).

Expressions of cytokeratin (CK) 5/6, p53 and Ki67 were investigated using horseradish peroxidase (HRP, Dako) immunohistochemistry. Briefly, paraffin-embedded tissue sections were cut at 4 µm thickness using microtome. The sections were placed on pre-coated slides and incubated in a hot air oven at 80°C for 40 minutes. For antigen retrieval, slides were placed in a heat-induced epitope retrieval Dako PT Link containing target retrieval solution (pH 9) with low (65°C) to high temperature (97°C) for 20 minutes, followed by cooling at 65°C and rinsing with phosphate buffered saline (PBS) thrice at pH 7.4. Slides were then placed in a humidity chamber and incubated with mouse monoclonal primary antibodies against CK 5/6 (D5/16 Dako, Denmark), p53 (DO-7 Dako, Denmark) and Ki-67 (MIB-1 Dako, Denmark) for 30 minutes at room temperature, followed by rinsing with PBS thrice. Subsequently, slides were incubated with HRP for 30 minutes at room temperature. After washing with PBS thrice, slides were treated with ready to use (RTU) chromogen/substrate (1:20) for 5 minutes, followed by washing with distilled water. Finally, the slides were counterstained with haematoxylin for 1 minute, washed with distilled water, dried and mounted. Positive control was performed with human OSCC, appendix and ovarian carcinoma for CK 5/6, Ki 67 and p53, respectively. The slides were examined under a light microscope and evaluated by a histopathologist. Images were captured using a camera (Nikon; eclipse-80i; Japan). The expression of CK 5/6 was analysed in the cytoplasm and p53 and Ki-67 were analysed in the nucleus [12–14]. The expressions were evaluated by determining the percentages of positively stained cells (brown in colour) in 1,000 cells (magnification, x400) with staining intensity graded as 0 (negative), 1 (weak), 2 (moderate) and 3 (strong). Ki-67 and p53 positive cells were scored in percentages as 1 (<30%), 2 (30–60%), 3 (>60%) and CK 5/6 as 1%–10%, 11%–50% and more than 50%.

Data were entered and analysed using GraphPad Prism version 9.3 (GraphPad software, San Diego, CA, USA) by log-rank test. *p*-value ≤ 0.05 was considered statistically significant.

## Results

### Macroscopic changes

**Figure 1.**
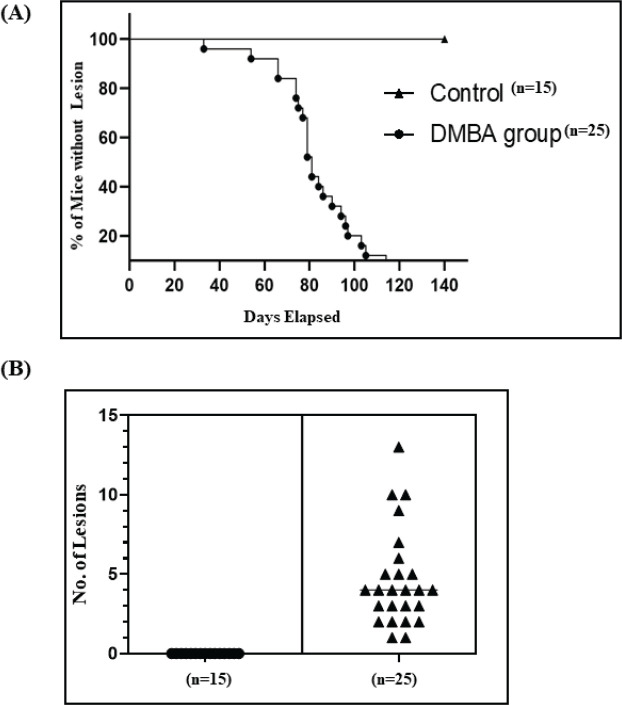
Graphical representation of DMBA-treated and control mice. (A): Number of days taken to develop lesions in the control and DMBA-treated mice. (B): Quantification of grossly visible tumours in DMBA-treated mice. *p-value ≤ 0.05.

In all mice undergoing the carcinogenic regimen, the earliest macroscopic changes were observed after 2 weeks of DMBA administration. These included hair loss in the cutaneous part of the lower lip, followed by the appearance of ulcerative or exophytic lesions at different weeks post-DMBA administration. All the 25 mice (100%) which underwent the accelerated carcinogenic regimen developed tumours/lesions. The earliest lesion was observed on day 33, while the highest number of days taken for development of a lesion was 126. The average number of days taken by our mice to develop lesion was 84.56 days. Controls that were not administered DMBA did not show any morphological changes throughout the course of study (Figure 1).

### Tumour burden

The overall average tumour burden was 4.64 lesions/mouse. The minimum number of lesions recorded in a mouse was 1 and the maximum number of lesions recorded was 13. For further analyses, lesions were divided into small, medium and large categories with ≤2 mm, 2.1–4 mm and >4 mm in diameter, respectively (Table 1).

At all times, a digital Vernier Caliper was used to measure the size of the lesions in two dimensions (length × width) in millimetres. Overall, the small category was the most commonly recorded lesion type. The smallest lesion was 0.87 mm in size, while the largest recorded lesion was 10 mm in diameter.

### Histopathological analysis

**Figure 2.**
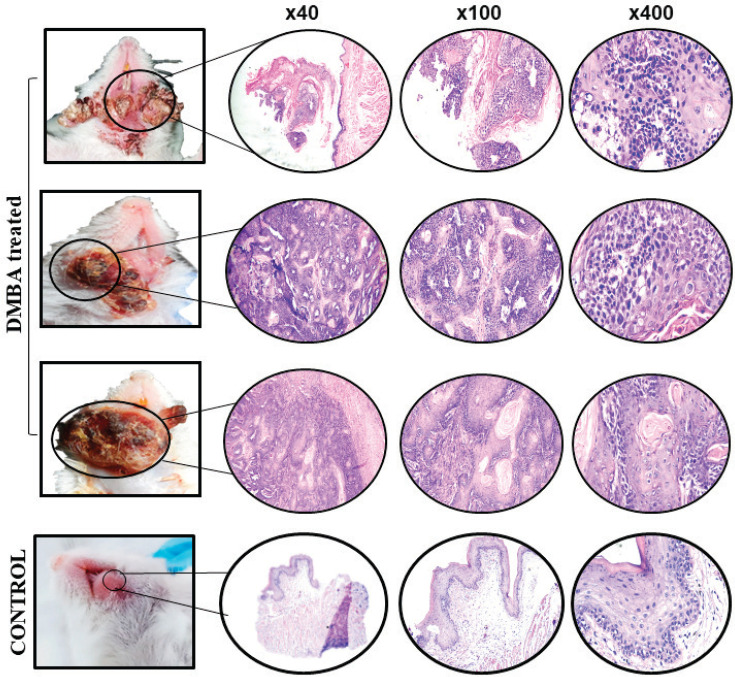
Gross (left) and microscopic (right) images of DMBA-treated versus control mice. Representative images of haematoxylin and eosin (H&E)-stained OSCC and control tissue sections at ×40, ×100 and ×400 magnification.

Of the 25 mice, a total of 3 were found dead on day 120, day 135 and day 137, respectively. Since these mice were found dead, their tissues were not histopathologically analysed. Nevertheless, tissues from these dead mice were morphologically evident as carcinomas. Tissues collected from the remaining 22 mice were investigated for microscopic analysis using haematoxylin and eosin (H&E) staining. Of the 22 tissues investigated, 21 were moderately differentiated OSCC and 1 showed only dysplastic features, which was characterised by full dysplastic epithelium with foci of invasion, as shown in Figure 2.

### Immunohistochemical analysis

**Figure 3.**
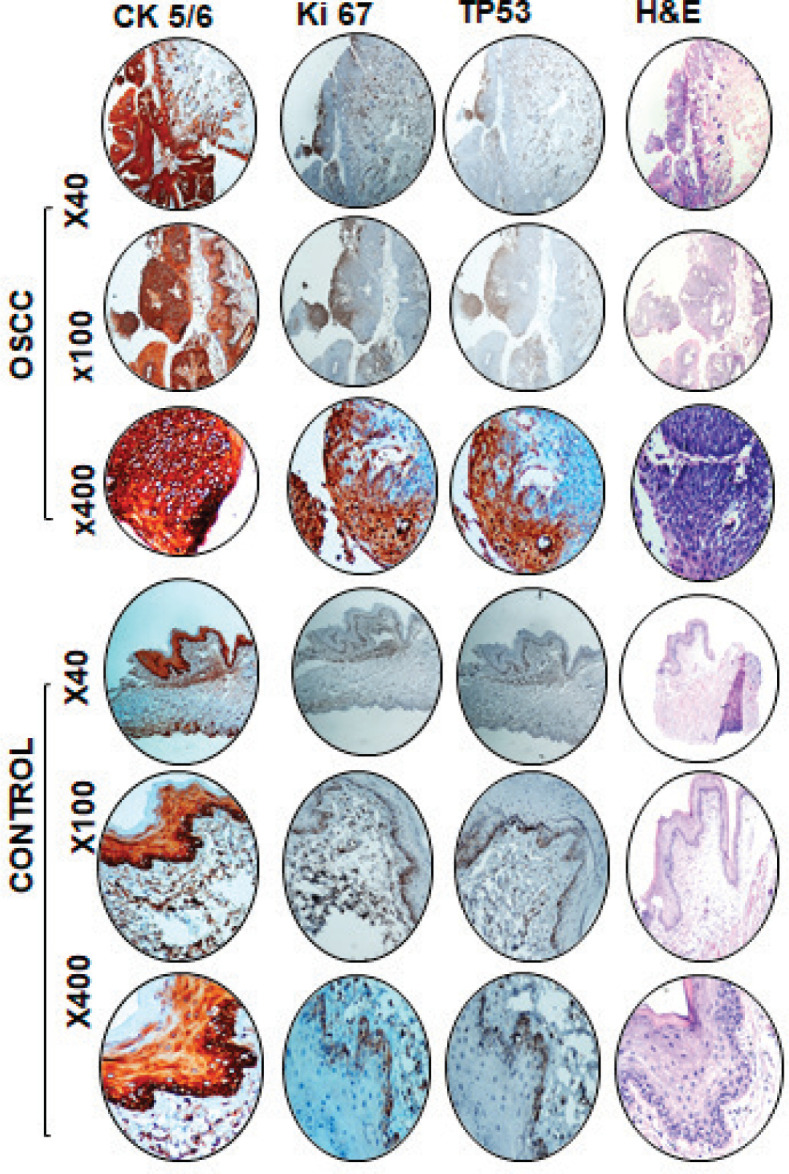
Representative images of immunohistochemical and haematoxylin and eosin staining in DMBA-treated and control mice. High expression of CK 5/6, Ki-67 and p53 in OSCC compared to control tissue at ×40, ×100 and ×400 magnification.

In order to confirm the epithelial origin of tumours, we investigated the expression of CK5/6 in murine tumours. Our data showed a significant expression of CK 5/6 in tumour tissues indicating epithelial origin of tumours developed due to DMBA treatment. The proliferative status of tumours was investigated using antibodies against Ki-67; a strong positive Ki-67 expression in tumours suggests high proliferative potential. We also investigated the p53 expression in cancer tissues and controls. Our data suggest high p53 expression in tumour cells compared to the controls, as shown in Figure 3.

## Discussion

Lip and oral cavity cancer is the second most common malignancy, with a 5-year prevalence of 14.78% and 9.1% mortality in Pakistan [1]. Recently, regional cancer registries reported a high burden of lip and oral cavity cancer in Karachi [2,3]. In Pakistan (South Central Asia), the majority of patients present in the late stages of cancer and despite treatment modalities, the overall 5-year survival rate is below 50% [15].

It is therefore highly relevant to develop a cancer model that can be exploited for diagnostic and therapeutic purposes. Histopathologically, the majority of lip and oral cavity cancers are OSCC [16]. Tobacco, whether smoked or chewed, is the major risk factor for OSCC [17]. Therefore, it is of prime significance to induce tumours using a carcinogen which is found in tobacco smoke. The compound DMBA is a polycyclic aromatic hydrocarbon present in smoked/chewed tobacco and is widely used to induce tumours [18,19].

One of the major reasons for slow progression in oral cancer-related research could be the unavailability of appropriate tools to test various drugs *in vivo*. The available rodent models of oral carcinogenesis are time-consuming (32 weeks) and *in vitro* studies, on the other hand, have their own limitations in terms of cost, lack of histological nature and lack of heterogeneity of tumour [20,21]. It is therefore highly relevant to devise a novel *in vivo* model that does not only exhibit accelerated carcinogenesis but is also close to human oral carcinogenesis.

With this context, we developed a murine model of accelerated oral carcinogenesis. We induced lip carcinogenesis via the topical application of DMBA. Several studies have reported the stages of chemically induced oral squamous cell carcinoma development [10,11]; however, the number of days taken to develop the first lesion and tumour size have not yet been reported. In our model, similar to human cancer, some mice developed lesion(s) earlier than others, as early as 5 weeks in 1 mouse while 15 (60%) mice developed exophytic lesions during 12 weeks and the 25 (100%) mice developed lesions by 18 weeks. Our findings are in contrast with previous study where red velvety rough appearance was observed after 4 weeks of DMBA but no macroscopic lesions (papilloma, ulcer and exophytic mass) were evident after 12 weeks. These differences could possibly be due to the differences in the animal strain and induction site, i.e., buccal pouch in hamsters, saliva and lymphocytic infiltrate that inhibited tumour development in a previous study model [22]. Another study revealed the first macroscopic change in tongue 19 weeks post-experiment and squamous cell carcinoma in 32 weeks in 4NQO-treated mice [20]. This could be explained based on the differences in application: 1 stroke of 4NQO in CBA mice, 3 times weekly for 16 weeks, as opposed to our regimen (detailed in the methodology).

In the carcinogenesis model described herein, the average time for onset of lesions was 12.08 weeks, which is in contrast to a previous study where the lesions developed in 21.1 ± 2.13 weeks [23]. The plausible reason for this difference could be the weight and age of mice. In our model, a young NMRI strain of mice, 6–8 weeks old, weighing 20 ± 2 g, were recruited compared to the previous study where Swiss mice, 14.2 weeks old, weighing 150 ± 30 g, were used.

We used Broder’s classification for grading OSCC [24]. Of 25 macroscopic lesions, 21 were diagnosed as moderately differentiated squamous cell carcinoma which was characterised by malignant neoplastic lesion arranged in nests and cords. Individual tumour cells showed individual cell keratinisation, vesicular nuclei, prominent nucleoli, abundant eosinophilic cytoplasm and keratin pearls. Stroma exhibited dense acute and chronic inflammation. These histopathological features mimic human OSCC [25] and one lesion showed dysplastic features. Three (12%) mice with tumours (macroscopic examination) were found dead; therefore, microscopic examination/diagnosis of their lesions was not performed.

In addition, immunohistochemistry was performed to further evaluate the similarity between mice and human OSCC. In our model, DMBA-treated mice demonstrated a strong positive (100%) expression of CK 5/6 in OSCC, indicating that the tumour was of epithelial origin. These findings are parallel with human OSCC [26]. TP53 is the most commonly mutated gene in human oral squamous cell carcinoma [27]. The mutated protein p53 accumulates in tumour cells and can be evaluated by immunohistochemistry. In our model, the expressions of p53 and Ki-67 were found to be strongly positive in DMBA-treated group compared to the control group; however, the positivity score was 1 (<30%). These findings are in accordance with other studies where the expressions of p53 and Ki-67 varied in both human and mice OSCC [28-31]. In summary, histopathological features and immunohistochemical expressions of CK 5/6, p53 and Ki-67 as described reveal that DMBA-treated NMRI mice developed tumours that closely mimic human OSCC. Nevertheless, the limitation of our study was that we did not evaluate the premalignant stages and lymph node metastasis.

## Conclusion

Taken together, we provide evidence for the development of an accelerated immunocompetent murine model of oral carcinogenesis which is close-to-human oral carcinogenesis. This model can be exploited as an important tool to understand the molecular circuitry of oral carcinogenesis and to investigate various biomolecules of diagnostic, therapeutic and prognostic significance.

## Conflicts of interest

The authors declare that they have no conflicts of interest.

## Funding

The authors received no financial support for the research, authorship and/or publication of this article.

## Authors’ contribution

SAS performed the experiment, acquired data, analysed and interpreted data and drafted the manuscript. MAQ conceived, designed and supervised the study, analysed and interpreted data and drafted the manuscript. SK, RK, BAK and JS facilitated sample collection and data acquisition. YS performed the histopathological analysis of the slides. All authors critically reviewed and approved the final draft and are responsible for the content and similarity index of the manuscript.

## Figures and Tables

**Table 1.** Tumour burden in DMBA-treated mice.

Mouse no.	Weight in grams	Small lesions ≤2 mm	Intermediate lesions 2.1–4 mm	Large lesions >4 mm	Total lesions
1	22 ± 2	0	1	2	3
2	22 ± 2	0	1	1	2
3	22 ± 2	0	2	0	2
4	22 ± 2	0	0	1	1
5	22 ± 2	0	0	1	1
6	22 ± 2	1	1	2	4
7	22 ± 2	1	1	0	2
8	22 ± 2	4	2	1	7
9	22 ± 2	0	3	1	4
10	22 ± 2	2	1	2	5
11	22 ± 2	4	1	0	5
12	22 ± 2	7	2	1	10
13	22 ± 2	3	1	1	5
14	22 ± 2	7	1	1	9
15	22 ± 2	10	1	2	13
16	22 ± 2	2	0	0	2
17	22 ± 2	7	1	2	10
18	22 ± 2	4	0	0	4
19	22 ± 2	1	2	1	4
20	22 ± 2	5	0	1	6
21	22 ± 2	3	1	0	4
22	22 ± 2	0	2	1	3
23	22 ± 2	1	0	2	3
24	22 ± 2	1	0	3	4
25	22 ± 2	0	2	1	3
		2.52	1	1.08	4.64
